# The interplay of sex steroid hormones and microRNAs in endometrial cancer: current understanding and future directions

**DOI:** 10.3389/fendo.2023.1166948

**Published:** 2023-04-21

**Authors:** Lovlesh Thakur, Sunil Thakur

**Affiliations:** ^1^ Department of Medical Microbiology, Post Graduate Institute of Medical Education and Research (PGIMER), Chandigarh, India; ^2^ Origin LIFE Healthcare Solutions and Research Center, Chandigarh, India

**Keywords:** microRNA, endometrial cancer, sex steroid hormones, estrogen, progesterone, hormone therapy

## Abstract

**Introduction:**

Endometrial cancer is a hormone-dependent malignancy, and sex steroid hormones play a crucial role in its pathogenesis. Recent studies have demonstrated that microRNAs (miRNAs) can regulate the expression of sex steroid hormone receptors and modulate hormone signaling pathways. Our aim is to provide an overview of the current understanding of the role of miRNAs in endometrial cancer regulated by sex steroid hormone pathways.

**Methods:**

A thorough literature search was carried out in the PubMed database. The articles published from 2018 to the present were included. Keywords related to miRNAs, endometrial cancer, and sex steroid hormones were used in the search.

**Results:**

Dysregulation of miRNAs has been linked to abnormal sex steroid hormone signaling and the development of endometrial cancer. Various miRNAs have been identified as modulators of estrogen and progesterone receptor expression, and the miRNA expression profile has been shown to be a predictor of response to hormone therapy. Additionally, specific miRNAs have been implicated in the regulation of genes involved in hormone-related signaling pathways, such as the PI3K/Akt/mTOR and MAPK/ERK pathways.

**Conclusion:**

The regulation of sex steroid hormones by miRNAs is a promising area of research in endometrial cancer. Future studies should focus on elucidating the functional roles of specific miRNAs in sex steroid hormone signaling and identifying novel miRNA targets for hormone therapy in endometrial cancer management.

## Introduction

Endometrial cancer (EC), a malignancy in the inner lining of the uterus, is a major contributor to deaths among gynecological malignancies. EC is characterized by abnormal cell growth, uncontrolled cell proliferation, excessive activation of signaling pathways, and microRNA (miRNA) activity (1). The progression and malignant transformation of EC are linked to central cellular processes such as epithelial-to-mesenchymal transition (EMT) and invasion (2). However, the pathogenesis of EC is complex and multi-factorial, and not fully understood. Studies have explored the influence of various factors in EC, such as estrogen receptor alpha (ER-α) activation of the MAPK signaling pathway (3) and the molecular responses in the endometrium to hormonal stimuli (4). Despite the growing use of molecular techniques in the study of gynecologic malignancies, the underlying molecular mechanisms and pathology remain elusive (5). There are concerns regarding the current guidelines pertaining to the diagnosis, prognosis, and treatment of EC, which are deemed inadequate (6). This highlights the need to explore innovative therapeutic strategies for this common female malignancy.

The role of non-coding RNAs (ncRNAs), such as miRNAs and long non-coding RNAs (lncRNA), in cancer diagnosis, prognosis, and therapy selection is gaining recognition in the scientific community. These ncRNAs play a key role in regulating cellular metabolism and transforming cells into cancer cells (7). Recent studies have identified miRNAs as potential therapeutic targets for EC (8). This review focuses on the role of miRNAs in diagnosing and treating EC, a type of cancer that primarily affects younger and post-menopausal women and is a major health concern. To highlight the most recent advances and gaps in this research area, articles since 2018 are included. This review examines various targets and pathways that may contribute to the development of EC by regulating miRNAs.

## Endometrial cancer

EC is the most generally diagnosed form of gynecological cancer in developed nations, making up approximately 5% of all cancers in women (9). EC originates in the endometrium, the inner lining of the uterus. It is most commonly found in post-menopausal women. EC has witnessed a marked surge in recent times, however, the underlying reason behind this trend remains elusive. EC is ranked as the fourth most common cancer in high-income countries and the most prevalent gynecological cancer globally, with 382,069 new cases reported in 2018 (10). In 2020, there were around 417,000 new cases globally and 97,300 deaths from EC, as per statistics (11).

Although, the exact causes of EC are unknown; lifestyle changes, obesity, infertility, hormone replacement therapy, and diabetes are believed to play a role. Advanced disease increases the likelihood of recurrence, and established risk factors include obesity and high estrogen exposure (12). EC is divided into type I (endometrioid) and type II (non-endometrioid). Type-I ECs are estrogen-dependent and have a good prognosis, while type II cancers are nonestrogen-dependent and have a more aggressive clinical course with a poor prognosis (13). However, recent methodologies have challenged this dualistic classification of EC (14). Further, it is found that Type I tumors have downregulation or mutations of the PTEN gene, while type II tumors have high expression of non-functional tumor proteins (7).

A confluence of several factors is believed to increase the likelihood of EC. Risk factors, such as obesity, diabetes, a family history of cancer, and Hormone Replacement Therapy (HRT), play a significant role in EC (15–17). Lifestyle factors like diet, physical activity, and smoking may also contribute to the development of EC (15, 16, 18–20). Additionally, certain medical conditions, such as polycystic ovary syndrome (PCOS), diabetes, and Lynch syndrome, may increase the risk of EC (21). EC is associated with abnormal production of hormones, inflammation, and hyperinsulinemia (22). The use of oral contraceptives reduces EC risk by 30-40% (23, 24). Coffee consumption, use of antidepressants, and male-origin microchimerism are linked to a reduced risk of EC (25–27), while the correlation between aspirin use and EC is inconclusive (28, 29). Smoking is inversely related to endometrial cancer, but the evidence does not support this relationship (18). The prognosis for early EC is generally good, but 7% of cases recur, and the 3-year survival rate is relatively low (30). This emphasizes the importance of conducting additional research into the molecular mechanisms of EC (such as miRNAs) for better prognosis and cancer prevention.

## MicroRNA

MicroRNAs or miRNAs or miRs are non-coding RNA molecules that play a crucial role in gene regulation, impacting cellular functions and associated with various pathological conditions, including EC. Their altered expression levels have been linked to prognostic factors and significantly impact tumorigenesis and progression in EC (31, 32). miRNAs have the potential to serve as non-invasive biomarkers for EC diagnosis by distinguishing between different conditions, such as endometrial hyperplasia (33, 34). They can regulate tumor suppressor genes and predict future EC development based on the patient’s immune microenvironment and insulin status (35, 36). miRNAs impact cell proliferation, differentiation, and apoptosis, and a higher accuracy miRNA signature can be obtained using miRNA clusters, serum type, and larger sample sizes (4, 37–39).

miRNAs can also act as oncogenes or tumor suppressors (40). The specific molecular characterization of miRNAs, involved in the regulation of the MAPK signaling pathway, has been shown to discriminate endometrioid endometrial carcinoma (EEC) from serous endometrial carcinoma (SEC) (41). An analysis of miRNA arrays revealed 20 miRNAs that differed significantly between patients with endometrial hyperplasia (EH) and those with simple hyperplasia/complex hyperplasia-non-atypical (SH/CH-nonA) (42). These findings highlight the important role of miRNAs in the regulation of gene expression and the pathophysiology of EC.

## Biogenesis of miRNA

**Figure 1:**
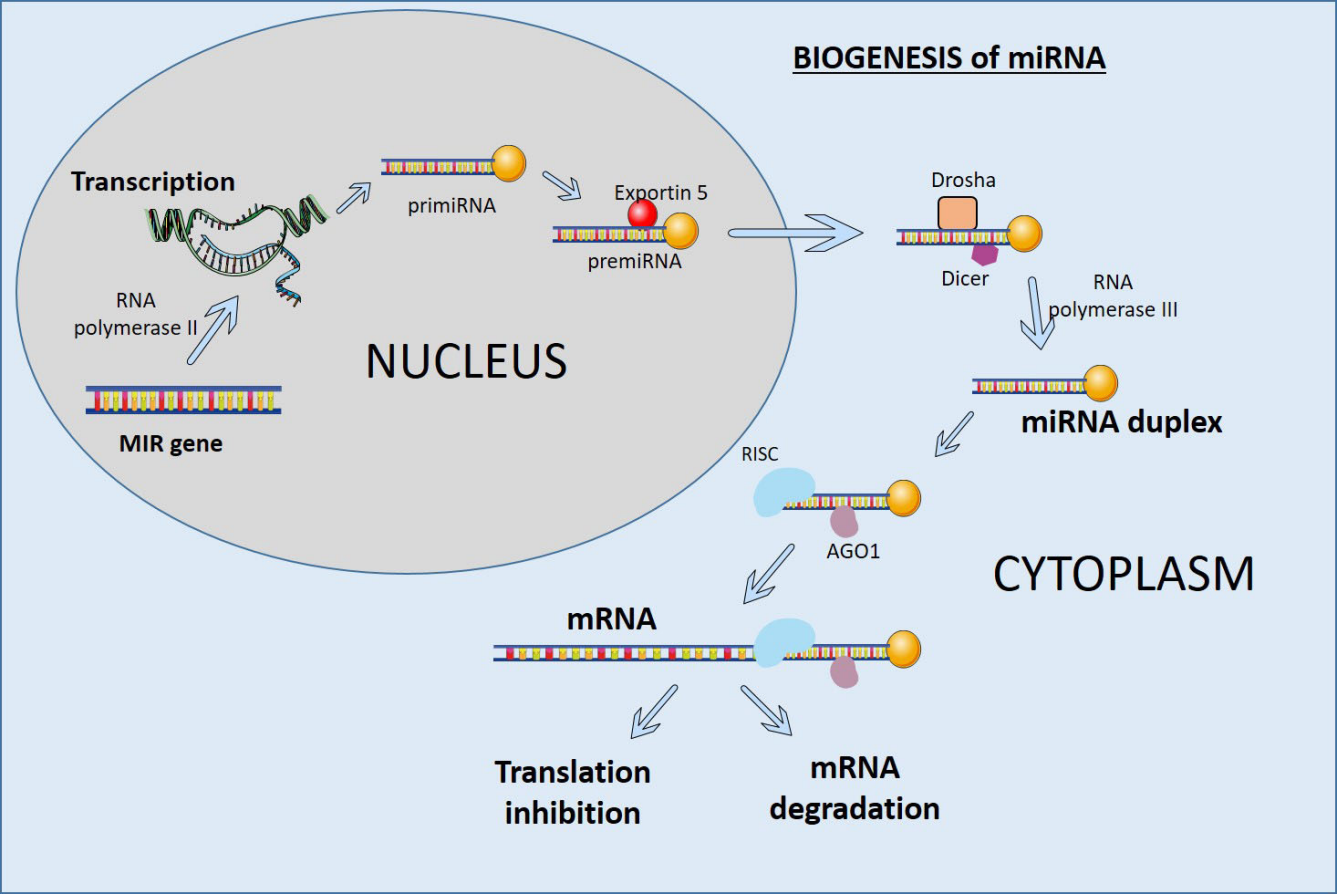
Mechanism of miRNA biogenesis and their functional implications.

miRNAs are small RNA molecules that play important roles in development, cell differentiation, and homeostasis. miRNAs are often present in tandem with multiple miRNAs in intergenic and intragenic genomic regions and are transcribed by RNA polymerase II as a single transcript (**Figure 1**). The RNase III enzyme Dicer processes them in the cytoplasm to produce mature double-stranded miRNAs. The processing of miRNAs can result in multiple sequence variants known as isomiRs. These isomiRs can form stem-loop structures and are cleaved by the RNase III enzymes Drosha and Dicer to generate precursor miRs and mature miRs. The primary precursor miRNA (primiRNA) is then transported from the nucleus to the cytoplasm by Exportin 5 and processed by Dicer to produce mature double-stranded miRNAs (43). These mature miRNAs are incorporated into the RNA-induced silencing complex (RISC) and target complementary sequences in the 3′ untranslated region (UTR) of mRNAs. By destabilizing target mRNAs, miRNAs reduce protein output and change gene expression (**Figure 1**) (44).

## Role of sex steroid hormones in endometrial cancer

Ovarian hormones, such as progesterone and estrogen, play a crucial role in regulating the endometrium and its monthly cycle. These hormones are responsible for the endometrium’s monthly proliferation, differentiation, and shedding and are the source of EC (45). The presence of Estrogen Receptor (ER) and Progesterone Receptor (PR) is linked to clinicopathological variables and can affect the prognosis of patients with EC. Steroid receptors ER and PR, activated by estrogen and progesterone, respectively, are critical in developing endometrioid-type EC (46, 47). These hormones are a key factor in EC occurrence and development, with 75-90% of EC incidents being dependent on estrogen (11). Estrogen exposure can result in endometrial hyperplasia and EC, while prolonged use of estrogens is associated with type 1 endometrioid malignancies (48). On the other hand, progesterone acts as an antagonist, inhibiting cell division, downregulating ER, and promoting cell differentiation (49). Overexposure to estrogen is a high-risk factor for EC, particularly for women undergoing estrogen-only hormonal therapy, using tamoxifen as an adjunct therapy, dealing with obesity, or suffering from PCOS. On the other hand, the progesterone receptor is a favorable prognostic marker for multiple solid tumors, but its expression is reduced in malignant tumors (50). The relationship between unopposed estrogen and EC is unclear, and progesterone can be used as a targeted therapy (51). Progesterone is used for treating young endometrial cancer patients, and the long non-coding RNA HOTAIR is a potential predictor for progesterone response. The expression of HOTAIR is regulated by LSD1, and inhibiting LSD1 can induce apoptosis (52). The use of oral contraceptives (OCs) and progestin-only contraceptives (POCs) has been linked to an increased risk of cancer (53, 54).

The loss of estrogen and progesterone receptors is associated with a higher rate of an advanced stage of endometrial cancer and increased expression of certain genes (55, 56). An imbalanced hormone state can arise from variations in the manifestation of genes and enzymes involved in the signaling of steroid hormones. Ovarian hormones regulate gene transcription by binding to DNA and initiating signaling pathways. It has been found that estrogen and progesterone are significant predictors of EC and that ER and PR are involved in genomic processes in cancer cells (57). Altered expression of hormone-regulated genes has been linked to a role for PAX2 in fine-tuning the interplay between ER alpha and PR (57, 58). Inhibiting ERRα/TGF-β can suppress *in vitro* invasion of EC cells, and ER signaling increases the expression of the oncogene PIWIL1 in ER-positive endometrial cancer cells (59, 60). Tumor suppressor genes such as CACNA2D3 are associated with EC and can exert a tumor-suppressive effect *in vitro* (3, 61). A novel signature of five genes has been developed to improve risk assessment and provide potential targets for EC therapies (11). Work is also being done to develop biomarkers for endometriosis, including the examination of PDCD4 and its regulation by progesterone (62). ER-positive endometrioid endometrial cancer (EEC) exhibits the highest rate of alterations in PTEN, while ARID1A is a common mutation in ER-negative EEC (63, 64). DNA methylation and hydroxymethylation also regulate gene expression and are associated with steroid receptors, enzymes involved in estrogen synthesis, and TET proteins that mediate hydroxymethylation (65).

The role of steroid hormones in EC mediated by insulin have also been investigated and findings showed the increased expression and phosphorylation of Akt, MAPK, and ERK in EC cells (66). Other factors include the regulation of transcription factors by cell adhesion, biomarkers in blood for hormone quantification, and intra-tumoral heterogeneity (67, 68). The immune system component NLRP3 inflammasome and the gene BCL11A have also been linked to EC progression, ERR, USP14, and estrogen (69–71). Additionally, the expression of the hormone receptors can also be used to predict lymph node involvement. A study found that high expression of ER has been associated with poor prognosis in endometrial tumors, and patients with ER/PR loss have deeper myometrial infiltration and higher rates of pelvic lymph node metastasis (72). One study found that decreased expression of combined ER/PR (estrogen receptor/progesterone receptor) was associated with a poorer outcome in endometrial cancer patients, but hormone receptor status alone did not significantly improve mortality prediction (73).

Variation in Hormone replacement therapy (HRT) type and the BMI status of the women is associated with an increased risk of EC (74). Obesity is a well-known risk factor for EC, but the mechanisms of obesity-related carcinogenesis are not well-defined and may vary based on the presence of Lynch syndrome (75). Hormonal therapy is considered best for low-grade disease and hormone receptor positivity, and it is better tolerated than chemotherapy (76). Another study found that oral estradiol plus vaginal progesterone therapy affected endometrial thickness, biopsy pathology, and cancer incidence in post-menopausal women (77). A study on the relationship of menopausal hormone therapy and EC found that patients with hormone therapy had lower BMIs, less diabetes, and fewer recurrences, but age and tumor stage had a bigger impact on overall survival (78). Prognostic factors such as the expression of ER, PR, and HER2 are also being assessed to improve patient outcomes (79). The cutoff values for the positivity of ER/PR in endometrial cancer are still being debated, and the expression of these receptors and other proteins are evaluated to determine their impact on EC prognosis (80).

The recurrence of EC is one of the major problem in its diagnosis and treatment. Researchers explored the role of sex hormones and insulin/insulin-like growth factor axis signaling in endometrial cancer recurrence. It was found that circulating estradiol and tumor tissue phosphorylated IGR1R/IR were associated with a higher risk of recurrence (12). A recurrence prediction model was created to anticipate the likelihood of endometrial cancer reoccurrence in stage I-II patients after surgical treatment. The final prediction model incorporated factors such as age, adjuvant treatment, histologic type, and expression levels of Ki67, ER, PR, and WFDC2 and showed adequate discrimination power (81).

## miRNA and EC

Multiple cancers can be identified and differentiated through miRNAs, serving as single biomarkers to diagnose and treat various gynecological cancers such as endometrial, breast, and ovarian cancer (82). miRNAs play important roles in pathological processes, indirectly regulating cell proliferation, differentiation, and apoptosis (35, 38, 39). The expression pattern of miRNAs may vary in EC (**Table 1**). Abnormal expression of miRNAs plays a critical role in tumorigenesis and progression in EC *via* significantly impacting PTEN levels (99). miRNAs can distinguish between different subtypes of EC, including endometrial hyperplasia, and have been shown to discriminate EEC from SEC (41).

**Table 1:** Targets and status of miRNAs specific to endometrial cancer.

miRNA	Targets	Endometrial Cancer	References
miR-200a	ALDH1A1, ABL1, CCND2	Up-regulated	(83)
miR-429	ALDH1A1, ABL1, CCND2	Up-regulated	(83)
miR-130	BHLHE40/41	Up-regulated	(84)
miR-107-5p	ERα	Upregulated	(85)
miR-21	–	Up-regulated	(86)
miR27a-5p	SMAD4	Up-regulated	(87)
miR-576-5p	ZBTB4	Up-regulated	(88)
miR-505	–	Up-regulated	(89)
miR-210-3p	RUNX1T1	Up-regulated	(90)
miR-18a-5p	THBD	Up-regulated	(91)
miR-34a	mMSET	Up-regulated	(92)
miR-424	mMSET	Up-regulated	(92)
miR-513	mMSET	Up-regulated	(92)
miR-205	–	Up-regulated	(93)
miR-223	–	Up-regulated	(93, 94)
miR-182	–	Up-regulated	(93)
miR-183	–	Up-regulated	(93)
miR-103	ZO-1	Up-regulated	(95)
miR-142-3p	–	Up-regulated	(96)
miR-146a-5p	–	Up-regulated	(96)
miR-151a-5p	–	Up-regulated	(96)
miR-215	LEFTY2	Up-regulated	(97)
miR-326	HuECSCs	Up-regulated	(98)
miR-181a	PTEN	Up-regulated	(99)
miR-27a	USP46	Up-regulated	(100, 101)
miR-150-5p	–	Up-regulated	(100)
miR-98	ABCC10/MRP-7	Up-regulated	(102)
miR-423	Bcl-2, E and N-cadherin, PTEN, AKT	Up-regulated	(103)
miR-200c-3p	–	Up-regulated	(104)
miR-652	RORA	Up-regulated	(105)
miR-501	HOXD10	Up-regulated	(106)
miR-21-5p	SOX17	Up-regulated	(107)
miR-15b-3p	KLF-2	Up-regulated	(108)
miR-200c	–	Up-regulated	(109)
miR-182-5p	PIAS1, STAT3	Up-regulated	(110)
miR-96-5p	PIAS1, STAT3	Up-regulated	(110)
miR-183	CPEB1	Up-regulated	(111)
miR-30c	mTA1	Up-regulated	(112)
miR-191	TET1	Up-regulated	(113)
miR-210	NFIX	Up-regulated	(114)
miR-141-3p	–	Up-regulated	(115)
miR-182	FOXF2	Up-regulated	(116)
miR-522	mAOB	Up-regulated	(117)
miR-449b-5p	mDM4	Up-regulated	(118)
miR-544a	mMP2, MMP9, Caspase 3	Up-regulated	(119)
miR-486-5p	mARK1	Up-regulated	(39)
miR-206	HDAC6	Up-regulated	(120)
miR-940	mRVI1	Up-regulated	(121)
miR-494-3p	PTEN	Up-regulated	(122)
miR-196a-5p	FOXO1	Up-regulated	(8)
miR-181c	PTEN	Up-regulated	(123)
miR-31a	–	Downregulated	(124)
miR-142	–	Downregulated	(124)
miR-125b	–	Downregulated	(124)
miR-101	–	Downregulated	(124)
miR-326	Bcl-2	Downregulated	(125)
miR-202	FGF2	Downregulated	(126)
miR-148b	DNMT1	Downregulated	(127, 128)
miR-135a	ASPH	Downregulated	(129)
miR-184	CDC25A	Downregulated	(130)
miR-195	TIMP2, MMP2, MMP9	Downregulated	(131)
miR-641	AP1G1	Downregulated	(132)
miR-31	–	Downregulated	(133)
miR-214-3p	TWIST1	Downregulated	(134)
miR-497-5p	–	Downregulated	(6)
miR-29a-3p	VEGFA/CDC42/PAK1	Downregulated	(135)
miR-20a-5p	Jak1/STAT3	Downregulated	(2, 136)
miR-449a	SRC	Downregulated	(137)
miR-23b	–	Downregulated	(138)
miR-29b	–	Downregulated	(31)
miR-23a	SIX1	Downregulated	(139)
miR-582-5p	AKT3	Downregulated	(140)
miR-218	ADD2	Downregulated	(141)
miR-302b-3p	ZEB1, Bcl-2, BAX	Downregulated	(142)
miR-302c-3p	ZEB1, Bcl-2, BAX	Downregulated	(142)
miR-302d-3p	ZEB1, Bcl-2, BAX	Downregulated	(142)
miR-379-5p	ROR1, ZO-1, E cadherin	Downregulated	(143)
miR-133b	SUMO1	Downregulated	(144)
miR-139-5p	HOXA10	Downregulated	(145)
miR-302a-5p	HMGA2	Downregulated	(146)
miR-367-3p	HMGA2	Downregulated	
miR-145-5p	DUSP6	Downregulated	(147)
miR-1290	EMT related proteins	Downregulated	(148)
miR-152	mMP10	Downregulated	(149)
miR-320a	IGF-1R, EIF4E, VEGFA, HIF1	Downregulated	(150–152)
miR-424	IGF-1R	Downregulated	(153)
miR-495	PIK3R1	Downregulated	(154)
miR-1271	LDHA	Downregulated	(155)
miR-381	IGF1R	Downregulated	(156)
miR-29c	mCL1	Downregulated	(157)
miR-101-3p	EZH2	Downregulated	(158)
miR-365	EZH2/FOS	Downregulated	(159)
miR-29b	–	Downregulated	(160)
miR-15a-5p	VEGFA	Downregulated	(161)
miR-543	TGF-β	Downregulated	(162)
miR-873	HTGF	Downregulated	(163)
miR-589-5p	TRIP6	Downregulated	(164)
miR-192-5p	IRAK1/NF-kB	Downregulated	(165)
miR-449a	NDRG1, LEF1	Downregulated	(166, 167)
miR-204-5p	–	Downregulated	(168)
miR-152	CDC25B	Downregulated	(169)
miR-205-5p	PTEN	Downregulated	(170)
miR-199a/b-5p	FAM83B	Downregulated	(171)
miR-34b	mYC, MET	Downregulated	(172)
miR-409	SMAD2	Downregulated	(173)
miR-340-5p	EIF4E	Downregulated	(151)
miR-137	–	Downregulated	(30)
miR-195	SOX4	Downregulated	(174)
miR-331-3p	WNT2	Downregulated	(175)
miR-200b-5p	WNT5A	Downregulated	
miR-146b-5p	NEAT1	Downregulated	(176)
miR-145	–	Downregulated	(177)
miR-143	–	Downregulated	

DNA methylation is a common phenomenon in cancer and suppresses the expression of miRNAs (60). Hypermethylation of miRNAs has been found in EC, and treatment with epigenetic inhibitors has been found to reduce cancer cell proliferation (178). The M6A RNA methylation modification is a pan-prognostic regulator of uterine cancer, with IGF2BP1 being the most important regulator (179). miRNAs influence the expression of tumor suppressor genes, and studies have shown that miRNAs related to the patient’s immune microenvironment and insulin status can predict the future development of EC (36). miRNAs have the potential to serve as novel, non-invasive biomarkers for EC diagnosis, with a miRNA signature that provides higher accuracy obtained by using miRNA clusters, serum type, and large sample sizes (4, 33, 37).

Multiple studies have found that miRNAs present in cluster significantly predict the prognosis and overall survival of EC patients. The miR-503 cluster, composed of six miRNAs, has been linked to endometrial endometrioid adenocarcinoma (EEA) (180). A nomogram based on age, clinical stage, and risk score calculated using a 4-miRNA signature showed high accuracy in predicting the overall survival of EC patients (181). A panel of 11 miRNAs effectively differentiated between ovarian and uterine serous carcinoma with 91.5% accuracy (182), and a 6-microRNA expression signature was established as a predictor for EC patient survival (183). A study found 4 miRNAs to be predictive biomarkers of overall survival among 561 differentially expressed miRNAs (184). A 26-miRNA signature was established as a crucial biomarker in predicting immunotherapy response (185), and a role was found for 21 miRNAs in predicting tumor mutation burden levels and regulating immune checkpoint expression (36).

Multiple miRNAs have also been evaluated as potential biomarkers for EC diagnosis in various biological samples such as urine, plasma/serum, exosomes, and endometrium. Urinary miRNAs, such as miR-205, -223, -182, -183, and -200a, have been up-regulated in EC patients, and their expression levels have the potential as a diagnostic test (93, 186). A six-miRNA signature in plasma has also shown superior performance in diagnosing EC (96). Exosomes derived from various sources, such as cancer-associated fibroblasts, tumor-associated macrophages, and tumor-infiltrating CD8^+^ T cells, hold the potential for EC diagnosis and treatment. Exosomes have unique miRNA expression profiles, with certain miRNAs, such as miR-148b and miR-320a, playing a role in cancer progression and others, such as miR-15a-5p and miR-192-5p, having the ability to distinguish EC patients from healthy subjects (104, 127, 165, 187).

The role of miRNA in stem cell growth has been demonstrated through *in vitro* analysis of human endometrial carcinoma stem cells (HuECSCs). It is shown that overexpression of miR-326 in HuECSCs significantly suppressed cell proliferation and progression through the cell cycle (98). miRNAs have been found to regulate genes associated with sensitivity to various anticancer drugs in EC. For example, MiR-98 and NEAT1 reduce drug resistance caused by MRP-7, an ATP-binding cassette transporter (102). MiR-135a is a potential predictor for EC chemotherapy response and prognosis (188). miRNA-29b has demonstrated the ability to inhibit cell proliferation and increase apoptosis in endometriosis cells (31). Overexpression of miR-365 has been found to enhance chemosensitivity to paclitaxel and mitigate cell migration and invasion (159). miRNA regulation in combination with anticancer drugs is a proposed treatment option to reduce EC expression (172). Therefore, miRNAs can be potential targets for future chemotherapy agents or cancer-specific biomarkers, as they are commonly involved in the development of various cancers, including breast, cervical, endometrial, ovarian, and vulvar cancer (189).

Most miRNA analysis in endometrial cancer is performed using qRT-PCR (96). Recently, TWAS has been proposed for identifying important miRNAs in disease (190). EC was also best predicted when PTEN loss and miR-200a were combined (42). Additionally, it has been demonstrated that a miRNA expression-based risk score is a standalone factor for forecasting the overall survival of EC patients (191). miRNA also exhibits differences in expression levels between different ethnic groups for EC. For example, miR-145 and miR-143 were found to be overexpressed in the Uygur group and downregulated in the Han group (192). Meanwhile, the upregulation of miR-223 was found to be a risk factor for death among African American and Caucasian patients with serous uterine carcinoma (94). Additionally, there were gender-specific differences in the interplay between cancer cells and the tumor microenvironment (193).

Recent research highlights the crucial role of the regulatory network between lncRNA-miRNA-mRNA (ceRNAs) in understanding the development of EC. This network identifies 169 prognosis-associated RNAs, including 92 lncRNAs, 16 miRNAs, and 61 mRNAs (194). Several genes, such as ADRA1A, ANGPTL1, FBXO32, KCNMA1, DCN, LEFTY1, LIN28A, LHX3, ST8SIA3, and CEP55, are also regulated by the lncRNA-miRNA-mRNA network (195). Moreover, miRNA-mRNA regulatory networks are linked to the biological process of negative regulation of transcription from the RNA polymerase II promoter and positive regulation from the RNAD promoter (166). Additionally, these networks have prognostic value, as low expression of some miRNA targets, such as LEF1 and NKD1, predicts advanced clinical stages and poor prognosis in EC patients (196). Furthermore, specific ceRNAs have been identified as modulators of the PI3K/AKT signaling pathway, which may suppress the growth and proliferation of tumor cells and could be potential targets for future treatments (170). Identification of 26 miRNAs and 66 target genes involved in various signaling pathways in endometrial tissue biopsies, providing potential targets for molecularly targeted EC therapies (83, 197). Changes in mRNAs and miRNAs also influenced the histaminergic system in endometrioid tissue, where miRNA 27a showed promise as a diagnostic biomarker for EC (100, 197). A review of 106 miRNAs found that miR-145 was the best predictor of EC, and it is widely recognized that miRNAs play important roles in pathological processes by functioning as either oncogenes or tumor suppressors (40, 198). Studies have explored the role of miRNAs in regulating the expression of tumor-related genes and showed that miRNAs could act as oncogenic or tumor suppressors (97, 113, 172, 199).

## EC and tumor suppressor miRNA

**Figure 2:**
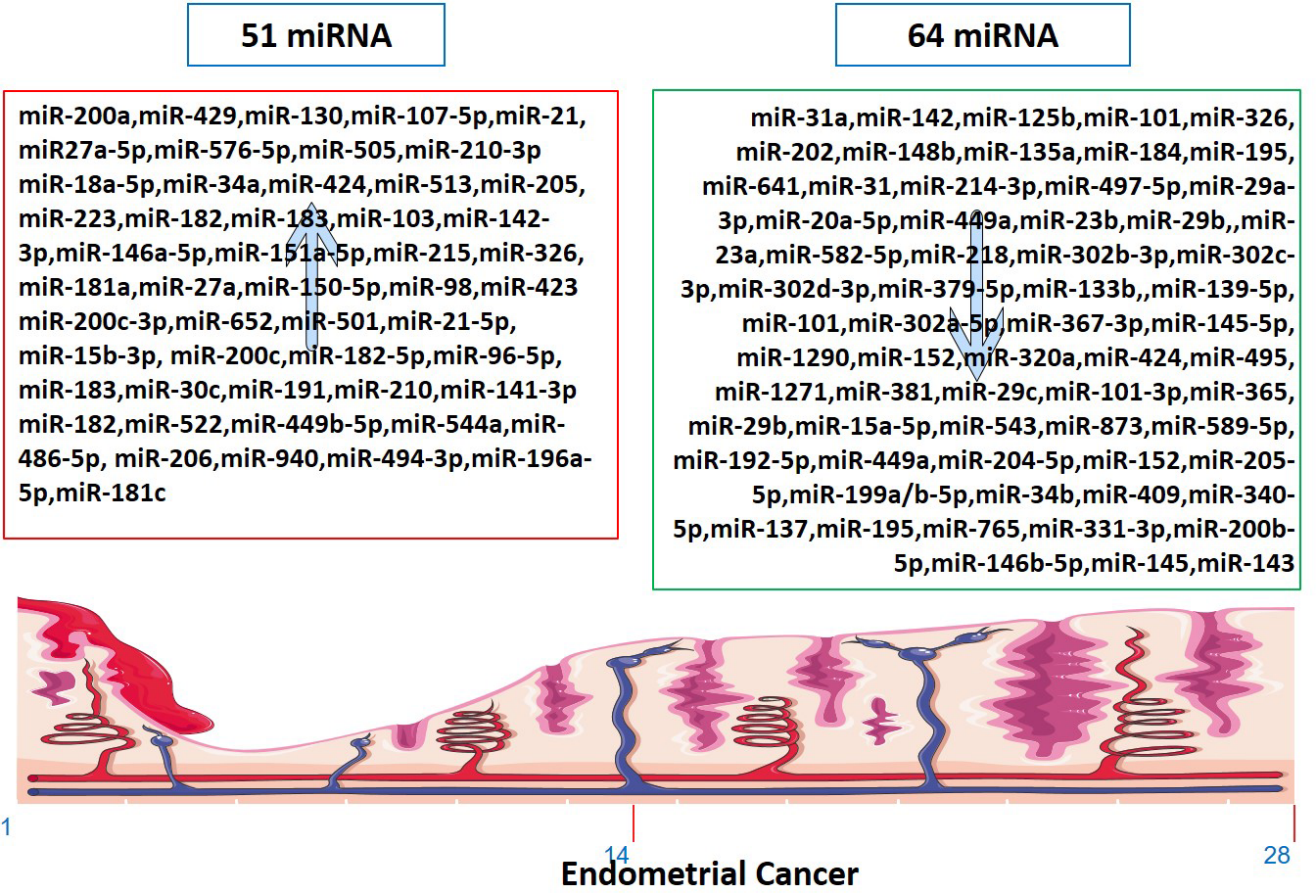
Up-regulated and down-regulated miRNAs in Endometrial Cancer. The markings 1, 14 and 28 in the bottom of endometrium, depicts the days of menstrual cycle.

Several miRNAs have been identified to be involved in the development and progression of EC, and it is established that miRNAs function as either oncogenes or tumor suppressors (**Figure 2**). The expression of miR-34a, miR-142, and miR-125b has been found to significantly decrease in endometrioid carcinomas with high proliferation, while miR-327, miR-202, and miR-214-3p have been shown to act as tumor suppressors in EC cells (124–126, 134). Downregulation of miR-23b, miR-125b-5p, miR-199a-3p, miR-221-3p, and miR-451a has been associated with EC (138). On the other hand, miR-652, miR-495, miR-1271, miR-381, and miR-29b have been identified as oncogenic miRNAs in EC (105, 154–156, 160). Furthermore, miR-15a-5p and miR-589-5p were tumor suppressors in EC cells (161, 164).

In cell lines studies, overexpression of miR-641, miR-184, miR-302a-5p/367-3p, miR-152, and knockdown of miR1290 and miR-873 had inhibitory effects on EC cell viability and proliferation (132, 146, 148, 149, 163). MiR-135a downregulation and miR-320a/miR-424 expression decreased EC cell proliferation and migration (129, 153). MiR-29a-3p inhibited EC cell proliferation, migration, and invasion by targeting VEGFA/CDC42/PAK1 signaling (135). MMSET played a pro-metastatic role in EC cell lines and tissues by repressing miR-34a, miR-424, and miR-513 (92). Downregulation of miRs-29-a, -b, and -c inhibited MCL1 apoptosis regulator BCL2 family member, MDM2 proto-oncogene, serum/glucocorticoid regulated kinase 1, sirtuin 1, and vascular endothelial growth factor A in HEC1A cells (157). MTA1 promoted cell proliferation, migration, and invasion abilities of EC cell lines and reversed the negative effect of miR-30c on EC cells (112). These findings provide insights into the potential roles of miRNAs as therapeutic targets in EC.

## EC and tumor-causing miRNA

As tumor-causing factors, several miRNAs have been identified as potential biomarkers and regulators of EC (**Figure 2**). miRNAs such as miR-130, miR-200a, miR-429, miR-107 5p, miR-21, miR-125b, miR-101, miR-27a-5p, miR-576-5p, miR-210-3p, miR-1903p, miR-18a-5p, miR-103, miR 215, miR-27a, miR-34a-5p, miR-146-5p, miR-423, and miR-302 have been shown to play different roles in promoting or suppressing EC progression by targeting various genes and signaling pathways (83–87, 90, 91, 95, 97, 101, 103, 124, 138, 200). These findings provide potential targets for diagnosis, prognosis, and therapeutic interventions in EC. miRNAs have been determined to impact the initiation and advancement of EC substantially. The expression of KLF2 in endometrial cancer cells is promoted by miR-15b-3p, while miR-210 negatively regulates NIFX expression. MiR-940 and miR-494-3p are overexpressed in EC tissues and cell lines, with MRVI1 and PTEN being identified as potential targets, respectively (114, 121, 122, 161). These miRNAs promote the proliferation, migration, and invasion of EC cells.

miRNAs also play a crucial role in regulating EC associated with lymph node metastasis. Downregulation of miRNA-184 is linked to lymph node involvement in low-risk EC patients (201). Overexpression of miR-501 can increase the activation of the AKT/mTOR pathway, leading to higher pelvic lymph node metastasis and reduced overall survival (106). Profiling the miRNA expression in EEC metastatic loci from lymph nodes could aid in identifying novel diagnostic markers and therapeutic targets (202). miR-204-5p is a tumor-suppressor miRNA associated with lymph node metastasis in EC, highlighting the importance of the lymph node status in determining adjuvant treatment (168). Finally, the expression of miR-15a-5p in EC is also correlated with lymph node metastasis, TNM stage, and mortality, and it was significantly reduced in EC patients who experienced recurrence or metastasis (203).

## miRNA and sex steroid hormone

Estrogen interacts with ER and GPER1 to control gene transcription and support cancer cell pathways. miRNAs negatively regulate gene expression and interact with estrogen in a complex way (34). MiR-205, -146a, and -1260b discriminate more than 90% of endometrial hyperplasia with atypia and endometrial intraepithelial neoplasia that progress to type I cancer (34). A worse prognosis of type II EC can be predicted by mRNAs linked to Wnt-signaling (196). MiR-22 and miR-206 target ER to prevent the progression of EC (198). miR-195 prevents EC invasion, migration, and EMT *in vitro* by targeting GPER1. On the other hand, miR-107-5p, miR-222-3p, and miR-205 promote tumor growth and migration *in vivo* as oncomiRNAs (198). Estrogen-regulated miRNA expression is specific to the cell and tissue and cannot be generalized to other species or tissues (4).

## Sex steroid hormones regulated miRNA

*In vitro* exposure of human endometrial stromal cells to estradiol increased the expression of miR-181b and let-7e while decreasing the expression of miR-27b (4). In EEC cell lines, estrogen treatment improved miR-200c and promoted miR-196a-5p expression, which targeted FOXO1 and increased cell viability (8, 204). On the other hand, progesterone inhibited cell-cycle and cell-viability in EC by regulating NEAT1/miR-146b-5p axis *via* Wnt/β-catenin signaling. It also induces miR-145/miR-143 to inhibit EC by targeting cyclin D2 and promoting miR-133a to increase endometrial cell proliferation (176, 177). It is also found that endometrial expression of miR-30b, miR-125b, miR-424, and miR-451 was lower in women with high blood progesterone levels compared to those with low blood progesterone levels (4). Moreover, miR-195 functioned as a tumor suppressor that targeted GPER1 to inhibit EC through PI3K/AKT signaling (131).

## miRNA regulation of sex steroid hormones

**Figure 3:**
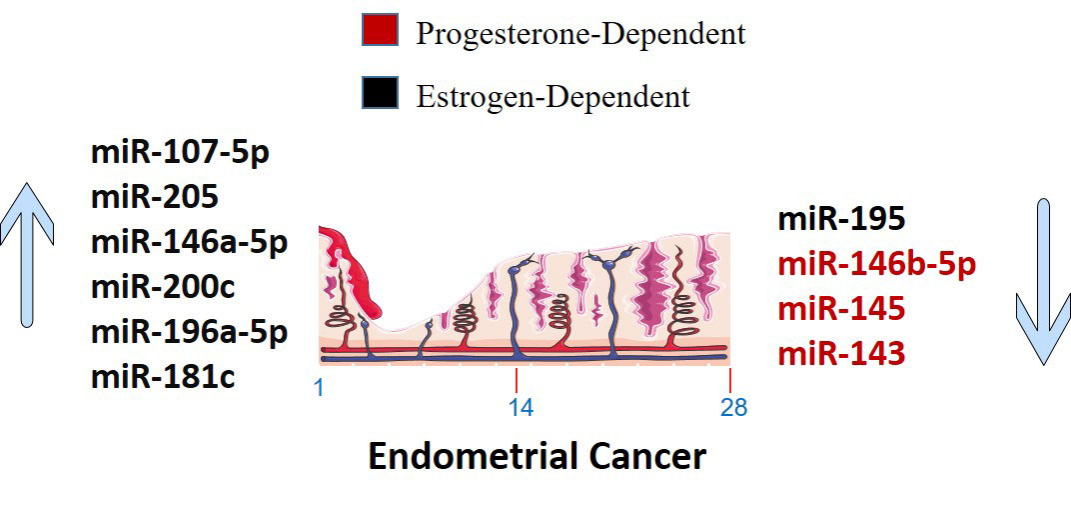
Sex steroid hormones dependent up-regulated and down-regulated miRNAs in Endometrial Cancer. The markings 1, 14 and 28 in the bottom of endometrium, depicts the days of menstrual cycle.

**Figure 4:**
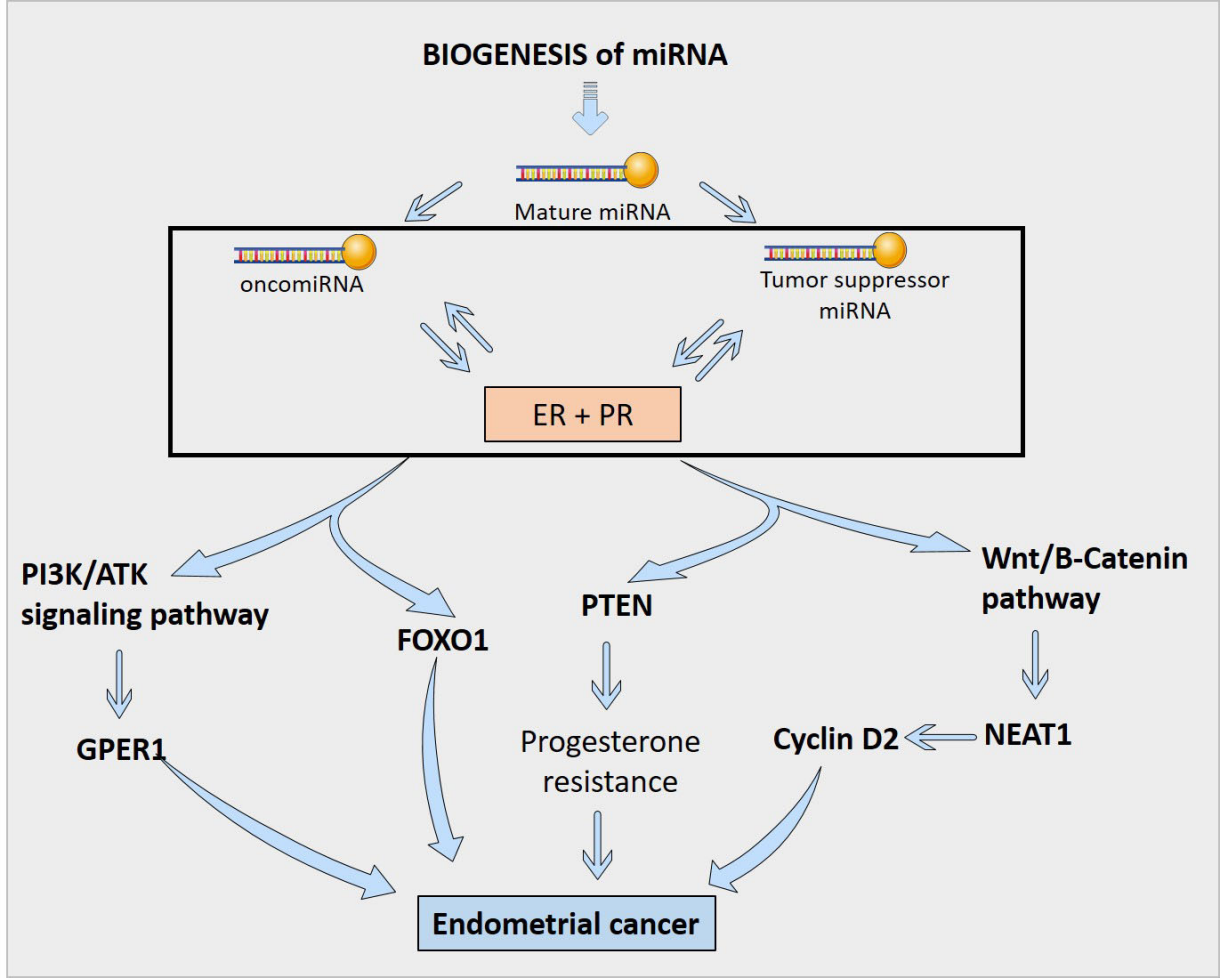
Interaction of miRNAs with sex steroid hormones in the regulation of Endometrial Cancer.

A number of miRNAs have been discovered to control the production of estrogen and progesterone receptors in endometrial cells (**Figure 3**). For example, miR-107-5p, which promotes tumor proliferation and invasion by targeting ERα, and miR-194-3p and miR-196a, which regulate the expression of PR-A and PR-B proteins (4, 85). Additionally, miR-181c affects estrogen-dependent EC cell growth by targeting PTEN, and miR-92a increases stromal endometrial cell proliferation and progesterone resistance (4, 123). However, the exact mechanisms by which miRNAs regulate ER in the endometrium are not yet fully understood (4). Although, in **Figure 4**, the possible mechanism by which sex steroid hormones can cause/suppress EC is depicted.

## Future directions

The regulation of sex steroid hormones by miRNAs or vice versa holds great promise for the future of EC research. The study of these interactions has the potential to uncover new mechanisms involved in the development and progression of EC, as well as provide new targets for therapeutic intervention. One area of future research could be to understand further the specific miRNAs involved in regulating sex steroid hormones in EC. This could be accomplished through large-scale sequencing studies or microarray analysis to identify miRNAs differentially expressed in EC compared to normal tissue. Additionally, functional studies could assess the effects of inhibiting or overexpressing specific miRNAs on hormone levels and EC cell proliferation. Another area of focus could be investigating the effects of miRNA-mediated regulation of hormones in EC under various conditions, such as in response to different treatments or stages of the disease. This information would be valuable in understanding the changing nature of miRNA control over hormones in EC and its potential as a therapeutic target. In conclusion, regulating sex steroid hormones by miRNAs is a highly promising area of research with numerous potential avenues for future exploration. As the field advances, we can expect a better understanding of the underlying mechanisms of EC and the development of new, more effective treatments for EC patients.

## Conclusion

miRNAs are tiny, non-coding RNA molecules that play crucial roles in regulating gene expression, including the regulation of hormones such as estrogen and progesterone. In endometrial cancer, imbalanced sex hormone levels contribute to tumor development and growth, making the study of miRNA-mediated regulation of these hormones a valuable avenue for research. Understanding the dual effect of miRNAs, i.e., the oncogenic and tumor suppressor effects, is warranted in EC. This knowledge can potentially lead to improved diagnosis and treatment options for EC patients.

## Author contributions

LT was responsible for conducting the literature review, collecting and analyzing data, and writing the first draft of the manuscript. ST contributed to the literature search, conceptualization and design of the study, provided critical feedback on the manuscript, and assisted with the editing and finalization of the manuscript. All authors contributed to the article and approved the submitted version.
